# Case Report: Proteomic analysis of cerebrospinal fluid in a retinoblastoma patient

**DOI:** 10.3389/fonc.2025.1511594

**Published:** 2025-04-24

**Authors:** Angela Galardi, Virginia Di Paolo, Chiara Lavarello, Ida Russo, Antonino Romanzo, Evelina Miele, Rita De Vito, Daniela Longo, Andrea Petretto, Franco Locatelli, Angela Di Giannatale

**Affiliations:** ^1^ Department of Pediatric Hematology/Oncology and Cell and Gene Therapy, IRCCS, Ospedale Pediatrico Bambino Gesù, Rome, Italy; ^2^ Core Facilities-Clinical Proteomics and Metabolomics, IRCCS, Istituto Giannina Gaslini, Genoa, Italy; ^3^ Ophthalmology Unit, IRCCS, Ospedale Pediatrico Bambino Gesù, Rome, Italy; ^4^ Department of Laboratories, Pathology Unit, IRCCS, Ospedale Pediatrico Bambino Gesù, Rome, Italy; ^5^ Neuroradiology Unit, Imaging Department, Bambino Gesù Children’s Hospital, IRCCS, Rome, Italy; ^6^ Catholic University of the Sacred Heart, Rome, Italy

**Keywords:** cerebrospinal fluid (CSF), retinoblastoma, proteomic analysis, liquid biopsy, case report

## Abstract

This study focuses on the proteomic analysis of cerebrospinal fluid (CSF) in a patient with stage III retinoblastoma (RB) with the aim to identify molecular changes associated with central nervous system (CNS) relapse. The child received systemic chemotherapy and intrathecal topotecan as CNS prophylaxis, along with enucleation of the left eye. After two chemotherapy cycles, CNS relapse occurred, evidenced by positive CSF findings and magnetic resonance imaging (MRI) showing leptomeningeal involvement at the anterior skull base. The child’s condition deteriorated, and two months later, he died due to progressive CNS disease. The aim of the study was to analyze serial CSF samples collected at different stages of treatment, as well as a control sample, to identify differences in CSF protein expression profiles during CNS RB relapse. Using mass spectrometry, a total of 1,029 proteins were identified across all CSF samples, samples were analyzed in duplicate ensuring technical replication. An unsupervised heatmap revealed 46 differentially expressed proteins. Over-regulated proteins in CSF-RB samples were primarily involved in inflammation, extracellular matrix remodeling, epithelial mesenchymal transition initiation, migration, invasion, and cellular metabolism (PON1, RNPEP, MCAM, NEGR1, NID1, SERPINA1, FAT2, RELN, NEGR1, and SEZ6). These processes are key drivers of cancer progression and metastasis. Proteomic analysis could be valuable in identifying proteins modulated in CSF during disease progression in RB patients, offering potential for new prognostic biomarkers.

## Background

Retinoblastoma (RB) represents the most common intraocular malignancy of infancy and childhood (1). Although the survival rates are over 95% for intraocular forms, in low-income countries the prognosis remains poor, ranging from 20% to 60% (2, 3). Among the dissemination routes, RB may spread to the central nervous system (CNS) through the optic nerve. This occurrence correlates with poor prognosis despite intensive multimodal treatment (4). In particular, in patients with advanced RB, CNS remains the predominant site of relapse. Thus, an early detection of CNS involvement is crucial to set up timely and tailored therapeutic approaches. The conventional diagnosis of CNS involvement relies on magnetic resonance imaging (MRI) and on the detection of tumor cells in CSF. Detection of biomarkers in the CSF could be useful in identifying patients at risk of developing CNS disease, even before the evidence of CNS involvement. In recent years, mass spectrometry has emerged as a sensitive tool in characterizing proteomic profiles of different biofluids, such as CSF, in neurological diseases and brain tumors (5–8). Importantly, the proteomic analysis of CSF may detect the presence of brain tumors before the onset of symptoms or imaging in animal models (9), reveling that CSF may be altered even at the earliest stages of malignancy. In this report, we perform a proteomic study of CSF in a patient affected by stage III (IRSS) RB in order to detect proteins changes occurring during CNS recurrence.

## Case presentation

### Clinical data

**Figure 1:**
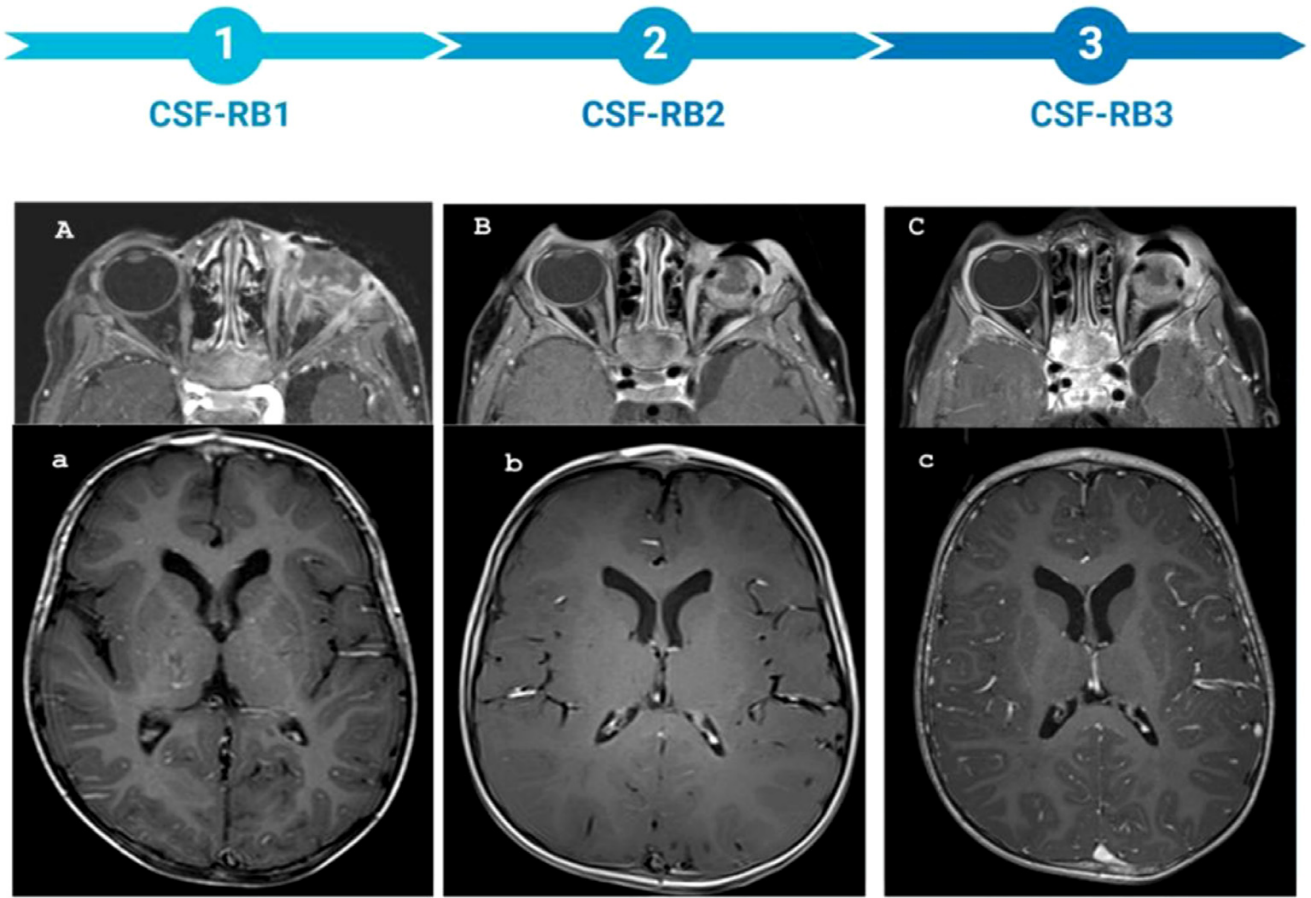
MRI of the patient throughout the entire treatment, along with the corresponding sample time points. **(A–C)** axial DIXON T1 post-CM: the timeline of optic nerve involvement. **(A)** presence of pathological tissue localized in the left retro bulbar site. This tissue takes the contrast medium (MDC) which localizes in the left retro bulbar region, is characterized by irregular profiles and extends to partially involve the extrinsic muscles, making their distal portion difficult to identify. The retro bulbar segment of the optic nerve is also partially affected, showing pathological enhancement. Additionally, there is pathological enhancement of the periorbital soft tissues. **(B)** Extensive involvement of the left optic nerve is evident following enucleation **(C)** Further progression of optic nerve involvement is observed, with irregular profiles of optic nerve. **(a–c)** axial T1w pst-CM: timeline of meningeal involvement. **(a)** no meningeal enhancement, **(b)** no meningeal enhancement, **(c)** intense meningeal enhancement.

An 8-month-old male child from Africa was admitted to the emergency department of the Bambino Gesù Children Hospital. He had a 6-months history of strabismus of the left eye associated with leukocoria diagnosed as cataract in his country and treated with eye drops. Due to the onset of exophthalmos, the child underwent MRI, which led to the diagnosis of RB (**Figures 1A, a**), and received 2 cycles of chemotherapy with etoposide and carboplatin. After his arrival at our hospital, the diagnostic workup documented a sporadic unilateral extraocular RB of the left eye, stage IIIa for orbit invasion, without CNS and bone-marrow involvement (**Figures 1B, b**). The patient underwent left eye debulking surgery (macroscopically incomplete enucleation) and received 3 cycles according to ICE schedule associated with intrathecal Topotecan at 0.3 mg per cycle. After 2 ICE cycles, the MRI and bone scintigraphy showed no local or CNS disease recurrence (**Figures 1 C, c**). However, the cytologic evaluation of CSF, performed during the third intrathecal injection of Topotecan, resulted positive for neoplastic cells. A new MRI showed impregnation of the leptomeninges of the anterior cranial bases confirming a disease recurrence at the CNS level. In light of these findings, the child received chemotherapy with Etoposide and Carboplatin for 5 days, in combination with intrathecal topotecan and thiotepa. CSF evaluation after chemotherapy was still positive for neoplastic cells. Due to incoercible vomiting and generalized hypotonia, the child was admitted to the emergency department where a Computerized Tomography (CT) scan showed massive CNS disease progression. The patient progressively worsened with cardiorespiratory arrest occurring 9 days after admission.

### Proteomic analysis

CSF samples were collected at the Bambino Gesù Children Hospital, Rome Italy. Parental informed consent was obtained for each patient. Three CSF samples were taken from a patient with RB (CSFRB) by lumbar puncture just before the injection of intrathecal Topotecan at the following time points: before starting chemotherapy (CSF-RB1), negative for neoplastic cells; after the 3th cycle of chemotherapy with Carboplatin, Etoposide, Ifosfamide (ICE), (CSF-RB2), positive for neoplastic cells; and just before starting chemotherapy with Etoposide and Carboplatin, 12 days after the previous sampling (CSF-RB3), still positive for neoplastic cells. We used one sample as normal control (CSFCTRL), collected from a 2-year-old boy with a history of post hemorrhagic tetra-ventricular hydrocephalus, during the peritoneal ventricular shunt procedure. All these samples were stored at −80°C until their use. For proteomic analysis, CSF was processed by in-StageTip (iST) method, samples were analyzed in duplicate ensuring technical replication (10). The tryp- column, thermostated at 55°C. The peptides were separated with an organic solvent at a flow rate of 250 nL/min, using a non-linear gradient of 5–45% solution B (80% CAN and 20% H2O, 0.1% FA) in 140 min, and analyzed using an Orbitrap Fusion Tribrid mass spectrometer (Thermo Scientific Instruments, Bremen, Germany). Orbitrap detection was used for both MS1 and MS2 measurements at re-solving powers of 120 K and 30 K (at m/z 200), respectively. Data dependent MS/MS analysis was performed in top speed mode with a 2 sec cycle-time, during which pre-cursors detected within the range of m/z 375−1500 were selected for activation in or-der of charge state, using CHarge Ordered Parallel Ion aNalysis (CHOPIN) (11). MaxQuant software was used to process the raw data, setting a false discovery rate (FDR) of 0.01 for the identification of proteins, peptides and PSM (peptide-spectrum match), a minimum length of 6 amino acids for peptide identification was required. Andromeda engine, incorporated into MaxQuant software, was used to search MS/MS spectra against Uniprot human database. All bioinformatics analyses were done with the Perseus software of the MaxQuant computational platform (12). Protein groups were filtered to require 70% valid values in total. The label-free intensities were ex-pressed as base log2, and empty values were imputed with random numbers from a normal distribution for each column, to best simulate low abundance values close to noise level. The Venn diagram of identified proteins was calculated using an online tool (13). For each comparison, a B significance test (Benjamini–Hochberg FDR <0.05) was used (14). To visualize the profile of the experiment, a hierarchical clustering of resulting proteins was performed on log2 intensities after z-score normalization of the data for each sample, using Euclidean distances.

## Results

**Figure 2:**
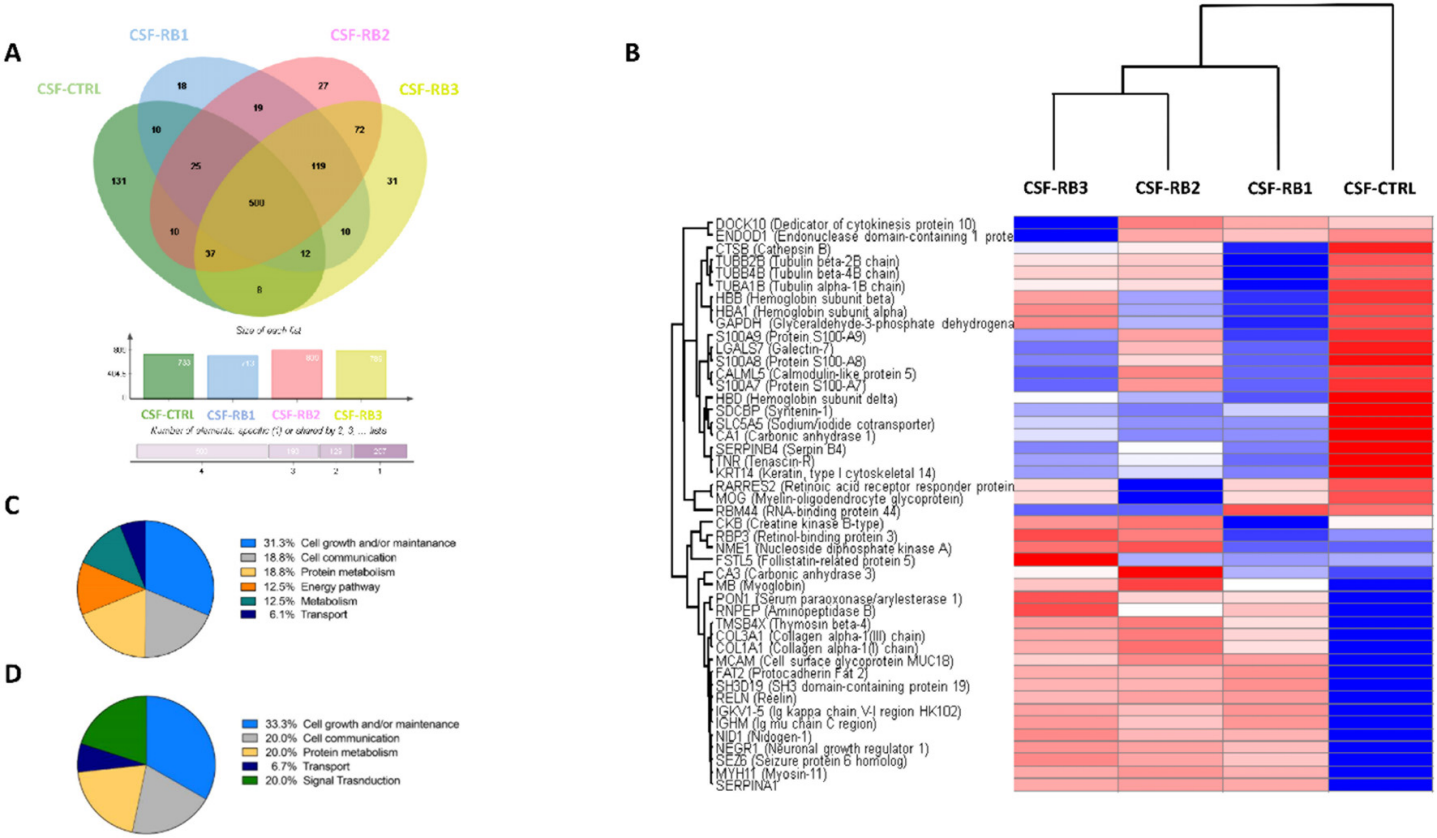
**(A)** Venn diagram showing a total of 1029 proteins detected in all CSF samples (CSF-CTRL, CSF-RB1, CSF-RB2, CSf-RB3), 500 proteins were in common in all samples. 733, 713, 809 and 789 proteins were found in CSF-CTRL, CSF-RB1, CSF-RB2 and CSF-RB3 respectively; 131, 18, 27 and 31 were exclusively present in CSF-CTRL, CSF-RB1, CSF-RB2 and CSF-RB3 respectively **(B)** Unsupervised hierarchical-clustered heatmap of proteins identified by significance B test. Heatmap showing the distinctive proteomic signature of the CSF-CTRL vs CSF-RB1, CSF-RB2, CSF-RB3. A red-blue scale corresponds to high and low protein amount, respectively. C-D) Graphical representation of biological processes gene enrichment obtained by FunRich software for low expressed **(C)** and high expressed **(D)** protein in CSF-RB (CSF-RB1; CSF-RB2; CSF- RB3) vs CSF-CTRL.

A total of 1029 proteins were identified in all CSF samples, of these 500 proteins were in common in all samples (**Figure 2A**). The Heatmap, resulting from the comparisons among the samples, showed 46 differentially expressed proteins (**Figure 2B**, **Table 1**). In all 3 CSF-RB samples we identified seven downregulated and seventeen upregulated proteins compared with the CSF-CTRL (**Figure 2B**, **Table 1**). By gene enrichment analysis performed by FunRich software (15), the downregulated proteins were mainly involved in cell growth, metabolism and cell communication, whereas the upregulated ones in cell growth, signal transduction and metabolism (**Figure 2C**). Four proteins were commonly and differentially expressed in CSF-RB2 and CSF-RB3 samples (positive for neoplastic cells). Specifically, 3 proteins were up-regulated [Creatine kinase B-type (CKB), Retinolbinding protein 3 (RBP3), Nucleoside diphosphate kinase A (NME1)] and one down-regulated [RNAbinding protein 44 (RBM44)]. Four proteins were down-regulated in CSF-RB1: Tubulin beta proteins (TUBB4B, TUBB2B, TUBA1B) and Cathepsin B (CTSB). Interestingly, FSTL5 was upregulated only in CSF-RB3, whereas 2 proteins (DOCK10 and ENDOD1) were down regulated in the same sample.

**Table 1:** Name of the protein in different samples.

Protein iD:	Gene names	Protein name:	Log_2_ LFQ intensity CSF-CTRL	Log_2_ LFQ intensity CSF-RB1	Log_2_ LFQ intensity CSF-RB2	Log_2_ LFQ intensity CSF-RB3
Q96BY6	DOCK10	Dedicator of cytokinesis protein 10	29,045	29,534	30,183	23,928
O94919	ENDOD1	Endonuclease domain-containing 1 protein	29,003	28,010	28,463	21,432
P07858	CTSB	Cathepsin B	28,176	24,084	26,330	25,965
Q9BVA1	TUBB2B	Tubulin beta-2B chain	29,654	19,240	26,846	26,271
P68371	TUBB42	Tubulin beta-4B chain	30,699	20,371	28,538	28,182
P68363	TUBA1B	Tubulin alpha-1B chain	32,414	25,396	29,989	29,694
P68871	HBB	Hemoglobin subunit beta	36,599	25,633	28,639	33,836
P04406-2	GAPDH	Glyceraldehyde-3-phosphate dehydrogenase	29,371	25,263	26,734	28,776
P06702	S100A9	Protein S100-A9	31,083	22,424	28,698	24,340
P47929	LGALS7	Galectin-7	29,505	21,580	26,298	21,876
P05109	S100AB	Protein S100-A8	31,113	21,533	26,365	22,277
Q9NZT1	CALML5	Calmodulin-like protein 5	28,726	21,486	27,294	21,293
P31151	S100A7	Protein S100-A7	29,831	21,444	27,569	21,486
P02042	HDB	Hemoglobin subunit delta	31,026	21,112	23,235	24,893
O00560-3	SDCBP	Syntenin-1	29,119	21,987	20,182	21,192
Q92911	SLC5A	Sodium/iodide cotransporter	30,229	25,120	25,111	25,955
E5RH81	CA1	Carbonic anhydrase 1	29,208	22,961	22,868	24,237
H0Y5H9	SERPINB4	Serpin B4	30,125	22,904	25,165	23,021
Q92752	TNR	Tenascin-R	28,814	23,889	25,439	24,507
P02533	KRT14	Keratin, type I cytoskeletal 14	28,630	23,949	24,956	24,252
Q99969	RARRES2	Retinoic acid receptor responder protein 2	28,268	25,756	19,299	25,576
H0Y8A0	MOG	Myelin-oligodendrocyte glycoprotein	28,955	26,302	20,425	26,374
Q6ZP01	RBM44	RNA-binding protein 44	33,283	33,889	27,367	27,408
P12277	CKB	Creatine kinase B-type	28,525	24,151	30,562	30,054
P10745	RBP3	Retinol-binding protein 3	23,091	20,279	31,394	33,047
P15531	NME1	Nucleoside diphosphate kinase A	24,967	24,967	29,114	28,742
Q8N475-2	FSTL5	Follistatin-related protein 5	24,168	23,949	24,179	28,581
P07451	CA3	Carbonic anhydrase 3	22,4192	24,417	30,802	26,262
P02144	MB	Myoglobin	22,280	27,401	31,331	28,714
P27169	PON1	Serum paraoxonase/arylesterase 1	26,037	28,935	28,998	30,206
A6NKB8	RNPEP	Aminopeptidase B	22,914	28,928	27,777	31,038
P62328	TMSB4X	Thymosin beta-4	25,470	28,952	30,031	29,653
P02461	COL3A1	Collagen alpha-1(III) chain	22,575	28,949	30,662	29,837
P02452	COL1A1	Collagen alpha-1(I) chain	26,150	30,203	31,641	30,864
P43121	MCAM	Cell surface glycoprotein MUC18	24,568	29,143	29,379	28,550
Q9NYQ8	FAT2	Protocadherin Fat 2	23,183	29,258	28,755	28,853
Q5HYK7-3	SH3D19	SH3 domain-containing protein 19	22,353	30,779	30,029	30,001
J3KQ66	RELN	Reelin	22,124	30,314	30,189	29,742
P01602	IGKV-1-5	Ig kappa chain V-I region HK102	22,456	29,532	28,789	29,271
P01871	IGHM	Ig mu chain C region	22,844	32,240	31,153	32,170
P14543	NID1	Nidogen-1	19,987	29,630	29,485	30,676
Q7Z3B1	NEGR1	Neuronal growth regulator 1	23,487	28,656	29,035	29,255
H0YF95	SEZ6	Seizure protein 6 homolog	24,099	28,292	28,668	29,003
P35749-4	MYH11	Myosin-11	22,953	30,134	30,640	30,278
A0A024R6I7	SERPINA1	SERPINA1	22,094	32,263	32,558	32,264

## Discussion

Although RB primarily affects the eyes, advanced disease mostly associated with delayed diagnosis, can lead to CNS involvement, including the brain and CSF, associated with extremely poor prognosis. Therefore, early detection of CNS involvement is crucial for allowing early therapeutic intervention. In RB, different biofluids, such as plasma, aqueous humor or CSF have been studied to detect tumor related biomarkers as prognostic predictors of disease evolution or treatment response (16). CSF samples from patients with non-metastatic RB and high-risk pathologic features showed that, among four cases with CSF relapse, two patients had GD2 synthase positivity in CSF as marker of minimal dissemination disease (MD) (17). In view of these findings, Laurent et al. suggested early and intensified intrathecal treatment for children with MD in CSF in order to improve the outcomes of these patients. Dimaras et al. also demonstrated that MD could be detected in patients with metastatic RB by using RB1 mutational analysis in CSF (18). Proteomic analysis involves the comprehensive study of proteins present in a biological sample. Since CSF is considered a primary route for metastatic dissemination (19), its proteomic analysis has been useful in improving the understanding of CNS tumors (20). Furthermore, proteins that are overexpressed in the CSF may serve as targets for developing new tailored treatment. For the afore mentioned reasons in this study, we have performed a proteomic evaluation of serial CSF samples in a patient affected by stage III RB with the aim to identify molecular changes occurring during treatment and CNS relapse. The differential analysis revealed a specific proteomics signature in CSF-RB samples. Among the upregulated proteins in CSF-RB, several have structural and physiological functions in the eye (COL1A1, COL3A1, TMSB4X, NID1) (21). NID1 has been described to promote tumor cell migration, invasion, epithelial-to-mesenchymal transition (EMT) and chemo-resistance in different tumors (22–24) similarly to MCAM (25). Other upregulated proteins are involved in neuronal/retinal development and cell migration (Fat2, RELN, NEGR1, SEZ6) (26–29). RELN is an extracellular matrix protein expressed during normal retinogenesis and seems to play an important role in regulating stem cell trafficking in neuronal and non-neuronal tissues (30). SEZ6 has been found be elevated in the CSF from patients with multiple sclerosis and inflammatory conditions (31), furthermore, has been shown to promote metastatic spread by affecting cellular adhesion, cytoskeletal and extracellular matrix remodeling and inducing EMT in cancer cells (32). Of note, RNPEP and PON1 progressively increased during treatment. RNPEP is a secreted enzyme belonging to M1 metallopeptidase family (33) and its circulating mRNA levels have been demonstrated to be an independent prognostic factor in different tumors (34). PON1 is an enzyme associated with high-density lipoprotein (HDL) and plays a role in detoxification processes (35); this protein has potential implications in ocular disorders associated with oxidative stress and inflammation (36–38). Similarly, SERPINA1, is an acute inflammatory protein involved in corneal defense and neuroinflammation (39). Among the down-regulated proteins in CSF-RB, we identified proteins involved in glucose transport (SLC5A) (40), synthesis of glycogen and lipids (CA1) (41), and exosomes biogenesis (SDCBP) (42); those proteins have been described to contribute to aggressive behavior of tumor via metabolic reprogramming and cell signaling. Other downregulated proteins were: protease inhibitor (SERPINB4), which may regulate the immune response against tumor cells (43), protein involved in brain development and synaptic plasticity (TNR) (44) and keratin (KRT14), which provides structural support and integrity to epithelial cells and is abnormal expressed in several tumors (45). The CSF samples that were positive for neoplastic cells, CSF-RB2 and -RB3, presented two commonly upregulated proteins involved in visual pathways: RBP3, which is involved in retinol transport (46), and CK-B, an enzyme that provides energy to photoreceptors for the visual cycle particularly expressed in retinal diseases (47, 48). Another protein, NME1, is involved in oxidative stress response (49) and its increased expression correlates with tumor aggressiveness in specific cancer types (melanoma, liver and breast) (50). CSF-RB3 showed two down-regulated proteins (DOCK10 and ENDOD1) and the upregulation of FSTL5. FSTL5 is a secretory glycoprotein expressed in restricted areas of CNS and few studies indicate its role on neo-plastic transformation and metastasis development (51, 52). DOCK 10 is also expressed in the brain and seems to have a role in neuroinflammation and cancer development by regulating cell adhesion and migration (53). ENDOD1 is a member of nucleases, which participate in DNA repair with abnormal expression in different tumors (54). Lastly, in the CSF-RB1 sample, which was negative for neoplastic cells, we found down-regulated three tubulin beta proteins (TUBB4B, TUBB2B, TUBA1B). Tubulins are microtubules subunits involved in cell shape maintenance (55). In particular, TUBB4B expression is correlated whit EMT initiation and affection of cell polarity (56) and TUBB2B is involved in visual development and function (57); mutations of TUBB4B and TUBB2B have been linked to several ocular disorders (57, 58).

## Conclusion

Overall, the proteomic analysis of CSF showed in our patient deregulation of proteins mainly involved in inflammation, extracellular matrix remodeling, EMT initiation, migration, invasion and cell metabolism. The CSF samples positive for neoplastic cells revealed proteins with a prominent role in visual pathways, including transport of retinol, photoreceptor’s function and oxidative stress response. The timepoint negative for tumor infiltration revealed several TUBB proteins that are related with cellular shape maintenance, cell polarity and EMT initiation. These results demonstrate how proteomic analysis of CSF can help to improve our understanding in RB. Further studies including large cohorts of patients with RB should be conducted to identify new prognostic biomarkers and potential therapeutic targets in this tumor.

## Data Availability

The datasets presented in this study can be found in online repositories. The names of the repository/repositories and accession number(s) can be found below: http://www.proteomexchange.org/, PXD056783.
